# Segmental Tracheal Resection for Thyroid Cancer: Perioperative Morbidity, Locoregional Control, and Survival

**DOI:** 10.1002/hed.70045

**Published:** 2025-10-08

**Authors:** Anastasios Maniakas, David C. Wilde, Isabelle Fournier, Emily K. Hyde, Li Xu, Jennifer R. Wang, Neil D. Gross, Erich M. Sturgis, Victoria Banuchi, Naifa L. Busaidy, Maria E. Cabanillas, Priyanka Iyer, Ramona Dadu, Steven G. Waguespack, Mimi I. Hu, G. Brandon Gunn, Michael Kwon, Salmaan Ahmed, Michelle D. Williams, Mark E. Zafereo

**Affiliations:** ^1^ Department of Head & Neck Surgery University of Texas MD Anderson Cancer Center Houston Texas USA; ^2^ Department of Otolaryngology‐Head and Neck Surgery Baylor College of Medicine Houston Texas USA; ^3^ Department of Endocrine Neoplasia and Hormonal Disorders University of Texas MD Anderson Cancer Center Houston Texas USA; ^4^ Department of Radiation Oncology University of Texas MD Anderson Cancer Center Houston Texas USA; ^5^ Department of Neuroradiology University of Texas MD Anderson Cancer Center Houston Texas USA; ^6^ Department of Anatomical Pathology University of Texas MD Anderson Cancer Center Houston Texas USA

**Keywords:** anaplastic, follicular, medullary, papillary thyroid cancer, poorly differentiated, segmental tracheal resection

## Abstract

**Background:**

Segmental tracheal resection is rarely needed for advanced thyroid cancer but is among the most complex, high‐risk thyroid surgeries.

**Methods:**

Retrospective study of patients undergoing segmental tracheal resection for thyroid cancer at MD Anderson Cancer Center (2005–2024).

**Results:**

We identified 120 patients with a median follow‐up of 4.6 years (range 0.02–16.38). Papillary thyroid cancer was most common (68%). The median number of tracheal rings resected was 4 (range 1–9). Seventeen (14%) patients had a new tracheostomy placed at the time of surgery, with 11 (9%) remaining trach‐dependent at last follow‐up. Twenty‐six (22%) patients had a return to the operating room within 30 days, while 3 (3%) patients suffered perioperative mortality. The more common postoperative complications included tracheostomy tube placement (10%), hematoma (7%), and anastomotic air leak (6%). Median hospitalization was 6 days (range 2–67). Locoregional control (LRC) and overall survival (OS) were 79% and 77% at 5 years, respectively.

**Conclusions:**

Segmental tracheal resection for advanced thyroid cancer is technically complex and high‐risk, but most patients stay recurrence‐free 5 years post‐surgery.

## 1 Introduction

Locally advanced thyroid cancer remains a clinically and technically challenging disease despite advances in understanding tumor biology and the development of novel systemic therapies. Surgical resection is generally first‐line therapy, with the goal of resecting all macroscopic disease [1]. Neoadjuvant systemic therapy approaches have more recently been considered for the most biologically aggressive histopathologies such as anaplastic thyroid cancer and poorly differentiated thyroid cancer [2, 3, 4, 5]. Following the strap muscles and recurrent laryngeal nerve, the trachea is the third most commonly grossly involved extrathyroidal structure, owing to its intimate anatomic relationship with the thyroid gland [6].

The extent of tracheal invasion of thyroid carcinoma can be classified into four stages of increasing tracheal involvement, as defined by Shin, Grillo, and colleagues [7]. Shave resection may be considered for tumors abutting the external perichondrium (Shin stage 1), or occasionally for tumors invading between cartilaginous rings (Shin stage 2). On the other hand, window or segmental resections are employed for disease extending through the cartilage (Shin stages 3 and 4), with the amount of trachea involved and the location of the disease being important factors in the type of tracheal resection and reconstruction. While window resections can be performed for limited anterior tumors, most patients with disease extending through the tracheal cartilage are managed with segmental tracheal resections [8, 9, 10].

Less than 1% of advanced thyroid cancer patients require segmental tracheal resection, but this procedure is amongst the most technically challenging and high risk in head and neck surgery [11, 12]. Ishihara and colleagues provided the first significant experience with segmental tracheal resection for 60 advanced thyroid cancer patients from 1973 to 1988, and this has remained the largest experience in the published literature to date [13]. While other series and systematic reviews have been published, the heterogeneity of both the extent of tracheal invasion as well as the type of surgical management [i.e., shave, window, or segmental (also known as circumferential or sleeve) resection] limit interpretation of these data [8, 14, 15]. This study describes the largest cohort to date of patients with locally invasive thyroid cancer requiring segmental tracheal resection, characterizing the oncologic and functional outcomes of this patient population.

## 2 Materials and Methods

### 2.1 Data Abstraction

Patients who underwent tracheal resection for advanced thyroid cancer at MD Anderson Cancer Center (MDACC) between January 01, 2005, and December 31, 2024, were retrospectively identified following institutional review board approval (PA14‐1082). This study was completed in accordance with the Declaration of Helsinki as revised in 2013. Patients were initially screened using CPT code 31785 (excision of tracheal tumor or carcinoma by transcervical approach) along with an international classification of diseases (ICD) code consistent with thyroid cancer. Patients who underwent segmental tracheal resection for thyroid cancer were included, while patients who underwent window resection, tracheal shave excisions, or concomitant total laryngectomy were excluded. All thyroid cancer histologies were included in an effort to report overall surgical outcomes, although histology‐specific subdivision is provided in subgroup analyses. Data was retrospectively extracted from electronic medical records, including demographics and histopathology, treatment and surgical history, intraoperative details, postoperative complications, adjuvant therapies, and recurrence and survival outcomes.

### 2.2 Definitions

Segmental tracheal resection was defined as a 360‐degree removal of the cervical trachea (at least one complete tracheal ring) for tumor extirpation, followed by primary reconstruction with end‐to‐end anastomosis. Temporary hypoparathyroidism was defined as iPTH less than 15 pg/mL (normal range 15–65 pg/mL) requiring supplementation with calcitriol or oral calcium for less than 6 months following tracheal resection. Permanent hypoparathyroidism was defined as postoperative iPTH less than 15 pg/mL requiring supplementation with calcitriol or oral calcium for more than 6 months following surgery. Permanent gastrostomy tube was defined as patients remaining gastrostomy dependent at 6 months postoperatively. Vocal fold mobility was assessed by documentation of preoperative flexible laryngoscopy. Postoperative flexible laryngoscopy was performed in patients with postoperative clinical suspicion of vocal fold paralysis. Anastomotic air leak (as a postoperative complication) was defined as patients requiring intervention beyond routine suction drain management. Patients were followed until their most recent clinic visit or death. Perioperative mortality was defined as death within 30 days of tracheal resection, or death prior to discharge from the hospital, or death in an outpatient skilled nursing facility following surgery.

### 2.3 End Points

Primary endpoints were overall survival (OS) and locoregional control (LRC). OS was calculated from the date of tracheal resection surgery to the date of last vital status, with patients censored at the date of last follow‐up or death. LRC was calculated from the date of surgery to the date of confirmed locoregional recurrence. Locoregional recurrence was determined from the review of imaging and pathology.

### 2.4 Statistical Analyses

Patient demographics, disease and surgical characteristics, treatment, and postoperative outcomes were summarized with descriptive statistics. The Kaplan–Meier method was used for time‐to‐event analysis, including OS and LRC. Median time to event in years with a 95% confidence interval was calculated. Pearson's chi‐squared test was performed to generate *p* values for comparison of the use of radiation over time. All statistical analyses were performed using SAS software, version 9.4, with *p* < 0.05 considered statistically significant.

## 3 Results

One hundred and twenty patients with thyroid cancer who underwent tracheal resection were included, with a median follow‐up from the day of surgery of 4.6 years (range 0.02–16.38) (Table 1). Fifty‐five patients (46%) had undergone prior thyroid surgery, including 23 patients (19%) having two or more prior thyroid/neck operations. Six patients (5%) had undergone prior neck external beam radiation, and 5 patients (4%) had neoadjuvant targeted therapy. Forty‐three (36%) had evidence of vocal fold hypomobility or immobility on pre‐operative flexible laryngoscopy. There were no instances of pre‐operative bilateral vocal cord dysfunction. Thirty‐seven (31%) patients had received prior radioactive iodine (median cumulative I131 activity 173 mCi; range 30.6–553 mCi). Fifty‐seven (48%) patients presented with concomitant distant metastases, while 22 (18%) others developed distant metastases subsequent to surgery.

**TABLE 1:** Demographics of 120 patients with segmental tracheal resection for advanced thyroid cancer.

Demographics	
Sex ‐ no. (%)	
Male	62 (52)
Female	58 (48)
Age ‐ years	
Median	60
Range (IQR)	34–89 (55–68)
Follow up ‐ years	
Median	4.6
Range (IQR)	0.02–16.38 (2.07–7.54)
Histology	
PTC	82 (68)
FTC	9 (8)
MTC	6 (5)
PDTC	15 (12)
ATC	8 (7)
Disease Status	
Primary	66 (55)
Recurrent	54 (45)
Distant metastasis^a^	57 (48)
Pre‐operative vocal fold dysfunction	
Unilateral	43 (36)
Bilateral	0 (0)
Prior treatment	
Tracheostomy	1 (1)
Gastrostomy	0 (0)
Neck external beam radiation therapy	6 (5)
Radioactive iodine	37 (31)

Abbreviations: ATC, Anaplastic Thyroid Carcinoma; FTC, Follicular Thyroid Carcinoma; IQR, Interquartile Range; MTC, Medullary Thyroid Carcinoma; PDTC, Poorly Differentiated Thyroid Carcinoma; PTC, Papillary Thyroid Carcinoma.

^a^ Distant metastasis at the time of presentation at our institution.

A median of 4 (range 1–9) tracheal rings were resected (Table 2; Figure S1). Forty‐five (38%) patients required resection of esophageal muscularis. There were 17/119 (14%) patients who had new tracheostomy tube placement at the time of tracheal resection and 12/119 (10%) who had a new tracheostomy tube in the post‐operative setting, not including one patient who had a tracheostomy tube before tracheal resection. Eleven (9%) patients remained trach‐dependent at last follow‐up. Eleven (9%) patients underwent free tissue transfer, most commonly as a free flap overlay of the tracheal closure. Free‐flap reconstruction was not associated with the length of resection or number of rings resected. Twenty‐seven (23%) patients had a pectoralis major (*n* = 6) or a sternocleidomastoid flap (*n* = 21) overlay.

**TABLE 2:** Surgical outcomes following segmental tracheal resection in 120 patients with advanced thyroid cancer.

Number of tracheal rings resected	
Median	4
Range (IQR)	1–9 (3.5–5)
Esophageal Muscularis Resection ‐ no. (%)	45 (38)
Tracheostomy	
At index operation	17 (14)
Most recent follow up	11 (9)
Gastrostomy tube	
New	26 (22)
Permanent	7 (6)
Reconstruction	
None	79 (66)
Pedicled muscle flap	30 (25)
Free tissue transfer	11 (9)
Length of hospital stay – days	
Median	6
Range (IQR)	2–67 (4–6)
Postoperative external beam radiotherapy	54 (45)
Postoperative radioactive iodine	40 (33)

Abbreviation: IQR, interquartile range.

No patients had intraoperative mortality. The most common complication was temporary hypoparathyroidism, which occurred in 54 (45%) patients in the postoperative period. Three (3%) patients developed permanent hypoparathyroidism. Thirty‐nine (33%) patients had new unilateral vocal fold immobility or hypomobility following tracheal resection. En bloc nerve sacrifice was necessary for tumor extirpation in the vast majority (31 of 39) of these patients. Four of the remainder had temporary neuropraxia, while the other four had permanent paralysis without nerve sacrifice. Twenty‐six patients (22%) returned to the operating room for postoperative complications within 30 days, most commonly for tracheostomy tube placement (*n* = 12), followed by hematoma (*n* = 8), infection (*n* = 6), and chyle leak (*n* = 2) (Table 3). The median length of hospital stay was 6 days (range 2 to 67 days).

**TABLE 3:** Postoperative complications and surgical morbidity following segmental tracheal resection in 120 patients with advanced thyroid cancer.

Complications and surgical morbidity	
Perioperative mortality ‐ no. (%)	3 (3)
30 day return to OR	26 (22)
New tracheostomy tube^a^	12 (10)
Hypoparathyroidism	
Temporary	54 (45)
Permanent	3 (3)
Wound infection	6 (5)
Hematoma	8 (7)
Chyle leak	2 (2)
Pneumothorax	3 (3)
Tracheoesophageal fistula	4 (3)
New vocal fold paralysis^b^	39 (33)
Anastomotic air leak	7 (6)

^a^ Placed in the post‐operative setting.

^b^ 31 had recurrent laryngeal nerve sacrifice at the time of surgery. Eight others had neuropraxia with intact nerve, of which four recovered function.

There were 3 (3%) perioperative deaths at 11, 67, and 72 days postoperatively. One of these patients had an acute cardiopulmonary event on post‐operative day 1 which may have been related to tracheal obstruction. Two other patients had significant medical comorbidities including muscular dystrophy and significant valvular heart disease and renal disease, with both patients suffering aspiration events during hospitalization and ultimately expiring during hospitalization and/or postoperative rehabilitation.

Forty (33%) patients were given adjuvant RAI. Fifty‐four (45%) patients received postoperative neck external beam radiation therapy (EBRT) with a median dose of 60 Gy (Range 30–66 Gy). When looking at rates of radiation delivery over the course of the study, 63% (*n* = 26/41) of patients treated up to and including 2013 received postoperative EBRT, while only 35% (*n* = 28/79) of patients treated after 2013 underwent EBRT (*p* = 0.003). Specifically, for differentiated thyroid carcinoma (DTC), in the pre‐2013 period, 56% (18/32) received postoperative EBRT, as compared to 24% (14/59) treated after 2013 (*p* = 0.002).

**FIGURE 1:**
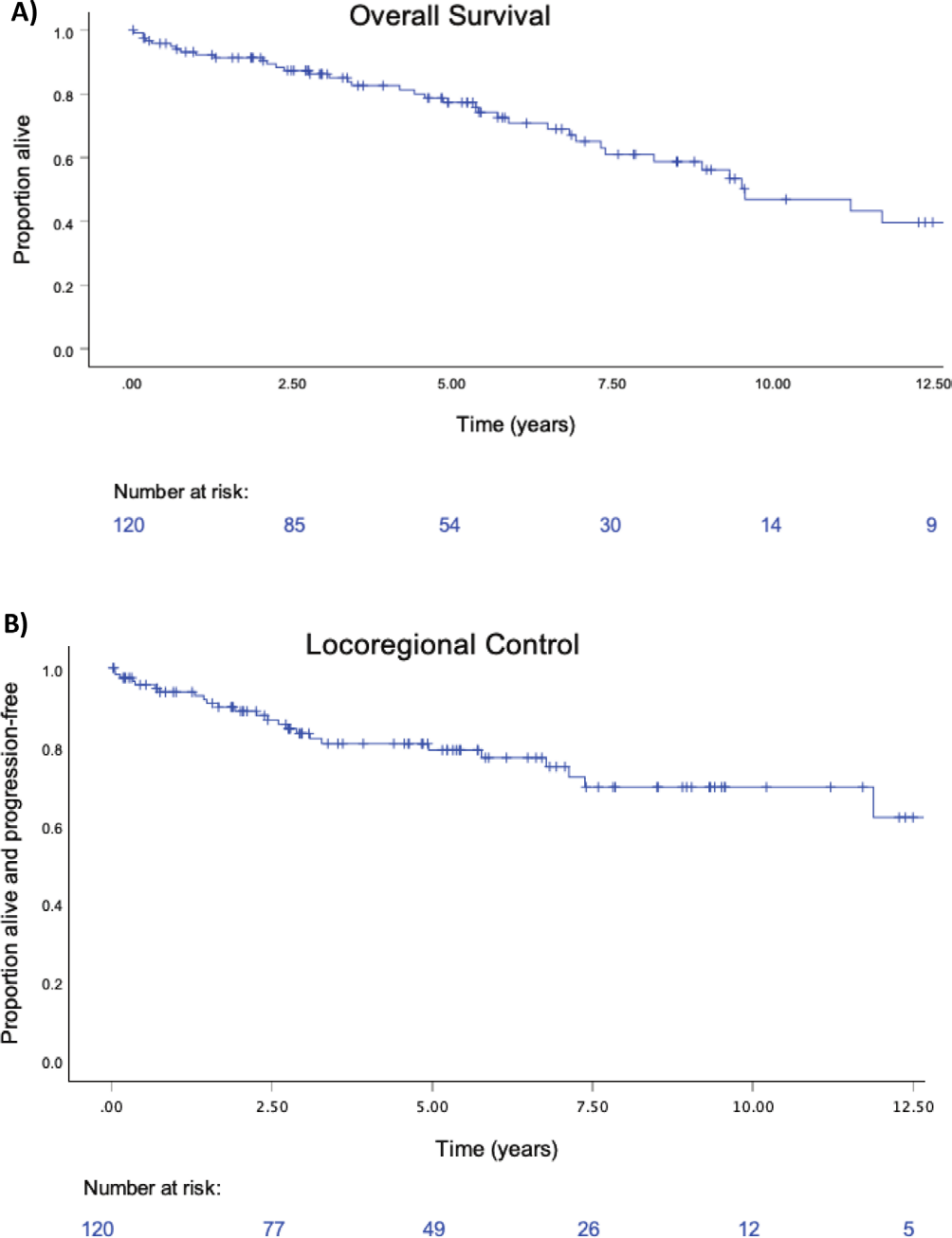
Overall survival (OS) of 120 patients with segmental tracheal resection for advanced thyroid cancer, with 1, 3, and 5‐year survivals 93.1% (95% CI = 88.5–97.7), 86.2% (95% CI = 79.7–92.8), and 77.3% (95% CI =68.7–85.8) respectively (A). Locoregional control (LRC) of 120 patients with segmental tracheal resection for advanced thyroid cancer, with 1, 3, and 5 years LRC 94.6% (95% CI = 90.4–98.8), 83.7% (95% CI = 76.2–91.6), and 79.3% (95% CI = 70.7–87.8) respectively (B). [Color figure can be viewed at wileyonlinelibrary.com]

Overall survival rates for the entire cohort (*n* = 120) were 93.1% (95% CI = 88.5–97.7) at 1 year, 86.2% (95% CI = 79.7–92.8) at 3 years, and 77.3% (95% CI = 68.7–85.8) at 5 years, while LRC rates at 1, 3, and 5 years were 94.6% (95% CI = 90.4–98.8), 83.7% (95% CI = 76.2–91.6), and 79.3% (95% CI = 70.7–87.8) respectively (Figure 1A,B). In total, 24 patients had a locoregional recurrence (21%), with 9 in central neck lymph nodes (8%), 11 in lateral neck lymph nodes (9%), and 4 involving the cricoid and/or trachea (site of previous tracheal resection) (3%). Specifically in patients with papillary thyroid carcinoma (PTC) (*n* = 82), OS rates were 95.1% (95% CI = 90.3–99.8), 87.7% (95% CI = 80.1–95.3), and 81.49% (95% CI = 71.7–91.3) at 1, 3, and 5 years, respectively, while LRC rates were 97.4% (95% CI = 93.8–100), 85.4% (95% CI = 76.5–94.4), and 80.68% (95% CI = 70.1–91.3), respectively (Figure S2A,B).

## 4 Discussion

We report the largest series to date of segmental tracheal resections for thyroid cancer, with the vast majority of patients alive and without locoregional disease at 3 years. These results are in the context of a patient population with significant treatment history including previous surgery (nearly 50%) and high preoperative morbidity/invasion of central neck structures (i.e., high rates of preoperative vocal cord paralysis and esophageal muscularis involvement). As described in the literature [14, 16], segmental tracheal resection for thyroid cancer remains a challenging surgery. In this cohort of 120 patients, we report relatively high rates of postoperative complications and return to the OR. Perioperative mortality was low but measurable. Overall, most patients remained tracheostomy‐free, feeding‐tube‐free, and disease‐free following surgery.

Adjuvant therapy was common in this cohort. With the emergence of numerous FDA‐approved targeted therapies for various subtypes of thyroid cancer over the past decade, there has been decreased reliance on postoperative EBRT. Significantly less postoperative EBRT was utilized in the overall study cohort, and amongst DTC patients specifically, in the 2014–2022 time period as compared to the 2005–2013 time period. Current institutional practice is to generally avoid postoperative EBRT for DTC patients after tracheal resection, as demonstrated by the relatively low rate of postoperative EBRT since 2014. A similar trend of foregoing postoperative EBRT is seen in patients with advanced medullary thyroid carcinoma, coinciding with increasing targeted therapy options for this patient population [17]. Patients with aggressive pathologies, such as ATC and PDTC, are more commonly recommended for postoperative EBRT. As targeted therapy options continue to evolve for patients with advanced thyroid cancer, it is likely that more patients will be treated with upfront neoadjuvant systemic therapy prior to surgery. However, thus far, the ability of neoadjuvant treatment to change the extent of surgical resection of the trachea has only been demonstrated in patients with BRAF*V600E*‐mutated anaplastic thyroid cancer [2, 3].

Approximately 24% of patients in this study required a tracheostomy at the time of surgery or in the post‐operative setting, over two‐thirds of which were subsequently able to be decannulated. When possible, tracheostomies should be avoided at the time of tracheal resection surgery as it may cause compromise to the structural integrity of the tracheal anastomosis and the potential introduction of saliva and bacteria into the surgical bed, possibly complicating postoperative recovery. This, in turn, may ultimately affect anastomotic integrity [18]. Factors that affect the likelihood of tracheostomy in association with tracheal resection included vocal cord status, length of tracheal resection, patient body habitus, and medical comorbidities.

In Ishihara et al.'s early description of 60 patients treated with segmental tracheal resection for thyroid carcinoma, only 34 patients had a complete resection of disease, with 87% OS in patients who had complete resection [13]. Their cohort had a bilateral vocal fold paralysis rate of 35%, which is significantly higher than more recent studies, including the current study, where none were reported, while they reported few other data regarding surgical complications. Piazza et al. performed a recent systematic review of 31 studies spanning from 1985 to 2021 comprising 656 patients (individual studies numbering 5–69 patients), wherein surgical complications occurred in 27%, with 4% permanent tracheostomy and 2% perioperative mortality [14]. The results of the current study reflect a similar rate of perioperative mortality, permanent tracheostomy, and overall post‐operative complications.

While others have previously reported on outcomes of tracheal resection for thyroid cancer, interpretations and comparisons can be limited by the heterogeneity of patient populations in various studies, specifically when it pertains to the type of tracheal intervention, as many studies include patients who underwent shave resections, window resections, or total laryngectomy with tracheal resection in the same analysis [8, 14, 15, 16]. The extent of surgical intervention in laryngotracheal procedures significantly influences patient outcomes, with less invasive techniques generally associated with more favorable prognoses. Patients undergoing less extensive tracheal procedures, such as shave resections or limited window resections, typically experience better outcomes. This improved prognosis can be attributed to two primary factors: firstly, these patients often present with less extensive disease, and secondly, the surgical approach itself entails lower complexity, resulting in a less complicated postoperative recovery. Conversely, patients requiring more extensive interventions, such as total laryngectomy combined with tracheal resection, represent a distinct clinical cohort. These cases differ markedly from those involving tracheal resection and reconstruction while preserving laryngeal function. The key distinguishing factor lies in the postoperative airway management; patients with an intact larynx following tracheal resection and reconstruction face unique challenges and risks related to airway complexity. This contrasts with the altered but potentially less complex airway management in laryngectomy patients.

Lastly, reinforcement of the surgical bed with a tissue transfer, either free or pedicled, is based on whether the patient has a history of prior EBRT, as tissue in the surgical bed is expected to have poorer healing and, subsequently, a higher risk of dehiscence of the tracheal anastomosis. Furthermore, if a large esophageal and/or skin defect is expected to be included in the resection, and/or there is to be major vessel exposure at the end of the resection, reconstruction is often recommended, usually in the form of a tissue transfer.

Limitations of this study include its retrospective study design and potential selection bias inherent to a tertiary cancer center. The selective use of post‐operative flexible laryngoscopy only for symptomatic patients may have led to underreporting of vocal fold mobility impairment. However, the study's strengths include the large cohort size and focus on a distinct group of patients undergoing segmental tracheal resection without laryngectomy. This cohort selection offers valuable insight into the complex multidisciplinary management of thyroid cancer patients with tracheal invasion who undergo larynx‐sparing surgery.

This study represents the largest single‐institution experience of segmental tracheal resection for locally advanced thyroid cancer. Findings demonstrate greater than 80% 3‐year locoregional control and overall survival. Most patients had previous surgeries and/or presented with invasion of additional central neck structures, including the recurrent laryngeal nerve and esophageal muscularis. The complexity of these cases is reflected in the elevated risk of perioperative complications observed in patients undergoing segmental tracheal resection. While most complications can be effectively managed, it is important to note that postoperative mortality, although rare, remains a potential risk. Over the past decade, there has been a notable decline in the use of adjuvant radiation therapy following segmental tracheal resection, coinciding with the emergence of targeted therapy options for this patient population. Given the advanced disease presentation and the intricate nature of both the surgical procedure and postoperative recovery, it is important that patients with tracheal invasion due to thyroid cancer be managed in high‐volume centers. Such centers should possess multidisciplinary expertise in advanced thyroid cancer to ensure optimal patient outcomes.

## Author Contributions

A.M.: conceptualization, methodology, formal analysis, writing – original draft preparation, writing – reviewing and editing. D.C.W.: formal analysis, data curation, writing – original draft preparation, writing – review and editing. I.F.: formal analysis, data curation, writing – original draft preparation. E.K.H.: formal analysis, data curation, writing – original draft preparation, writing – review and editing. L.X.: methodology, formal analysis, writing – reviewing and editing. J.R.W.: writing – reviewing and editing. N.D.G.: writing – reviewing and editing. E.M.S.: writing – reviewing and editing. V.B.: writing – reviewing and editing. N.L.B.: writing – reviewing and editing. M.E.C.: writing – reviewing and editing. P.I.: writing – reviewing and editing. R.D.: Writing – Reviewing and Editing. S.G.W: writing – reviewing and editing. M.I.H: writing – reviewing and editing. G.B.G: writing – reviewing and editing. M.K.: writing – reviewing and editing. S.A.: writing – reviewing and editing. M.D.W.: writing – reviewing and editing. M.E.Z.: conceptualization, methodology, formal analysis, writing – original draft preparation, writing – reviewing and editing.

## Disclosure

A.M.: Contracts for pre‐clinical trials with Jazz Pharmaceuticals. M.E.Z. reports placement on the Thyroid International Recommendations Online international advisory board. The other authors have nothing to report.

## Ethics Statement

This study was conducted in accordance with the ethical principles outlined in the Declaration of Helsinki.

## Supporting information


**Data S1:** Supporting Information.

## Data Availability

The data that support the findings of this study are available from the corresponding author upon reasonable request.
