# Supplementary material for: Segmental Tracheal Resection for Thyroid Cancer: Perioperative Morbidity, Locoregional Control, and Survival
Source: Head Neck. 2025 Oct 8;48(3):644–9. doi: 10.1002/hed.70045 (PMC12891758; doi:10.1002/hed.70045)

**Suppl Figure 1.** Frequency of tracheal ring resection according to tracheal ring number

**Suppl Figure 2.** Specifically in patients with papillary thyroid carcinoma (PTC) (n=82), OS rates were 95.1% (95% CI = 90.3-99.8), 87.7% (95% CI = 80.1-95.3), and 81.49% (95% CI = 71.7-91.3) at one, three, and five years, respectively, while LRC rates were 97.4% (95% CI = 93.8-100), 85.4% (95% CI = 76.5-94.4), and 80.68% (95% CI = 70.1-91.3), respectively.

**A)**


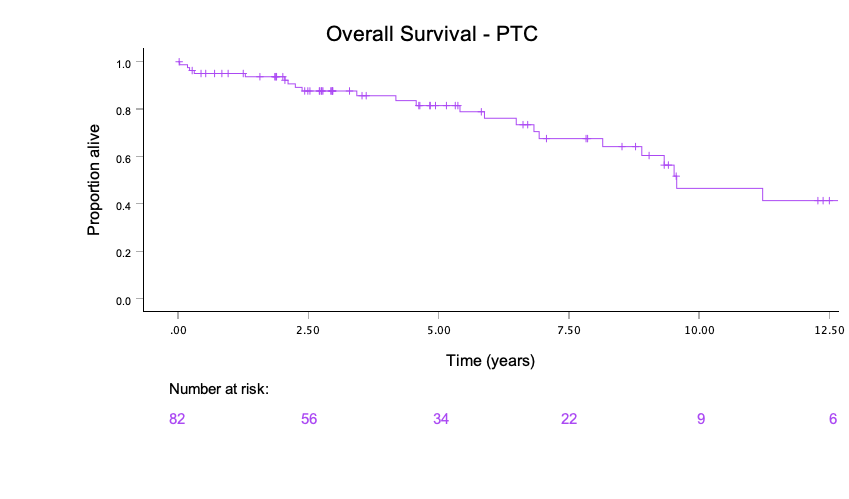


**B)**


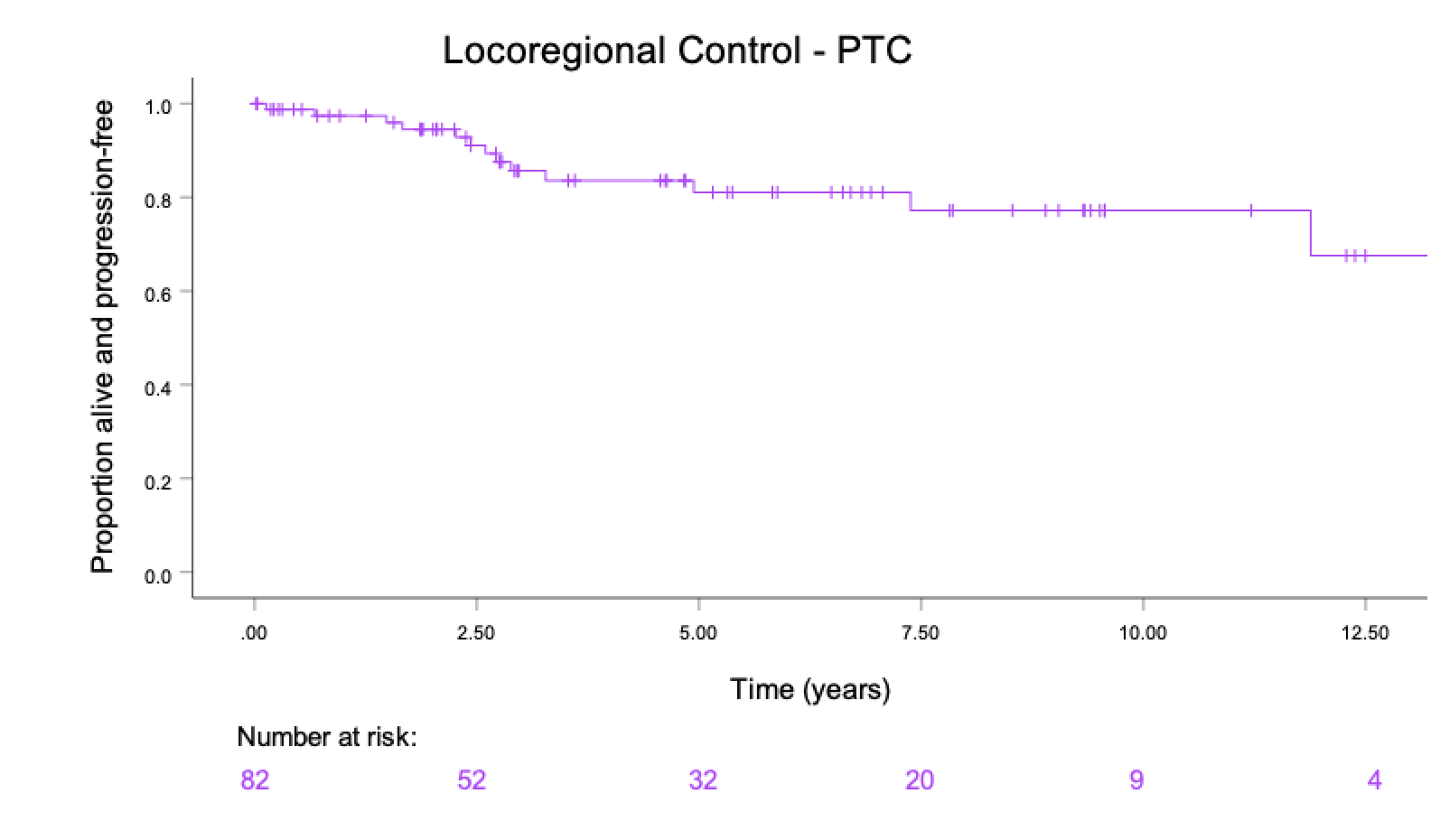

Supplement: Supplementary file 1 — Data S1: Supporting Information. [file HED-48-644-s001.docx]
